# Predicting Cognitive Decline in Parkinson’s Disease Using Artificial Neural Networks: An Explainable AI Approach

**DOI:** 10.3390/brainsci15080782

**Published:** 2025-07-23

**Authors:** Laura Colautti, Monica Casella, Matteo Robba, Davide Marocco, Michela Ponticorvo, Paola Iannello, Alessandro Antonietti, Camillo Marra, for the CPP Integrated Parkinson’s Database

**Affiliations:** 1 Department of Psychology, Università Cattolica del Sacro Cuore, 20123 Milan, Italy; matteopaolo.robba@unicatt.it (M.R.); paola.iannello@unicatt.it (P.I.); alessandro.antonietti@unicatt.it (A.A.); camillo.marra@unicatt.it (C.M.); 2 Natural and Artificial Cognition Lab “Orazio Miglino”, Department of Humanistic Studies, University of Naples “Federico II”, 80138 Naples, Italy; monica.casella@unina.it (M.C.); davide.marocco@unina.it (D.M.); michela.ponticorvo@unina.it (M.P.); 3 Area of Economic and Business Education, University of Mannheim, 68161 Mannheim, Germany

**Keywords:** Parkinson’s disease, cognitive decline, machine learning, artificial neural network, explainable AI, prevention

## Abstract

Background/Objectives: The study aims to identify key cognitive and non-cognitive variables (e.g., clinical, neuroimaging, and genetic data) predicting cognitive decline in Parkinson’s disease (PD) patients using machine learning applied to a sample (*N* = 618) from the Parkinson’s Progression Markers Initiative database. Traditional research has mainly employed explanatory approaches to explore variable relationships, rather than maximizing predictive accuracy for future cognitive decline. In the present study, we implemented a predictive framework that integrates a broad range of baseline cognitive, clinical, genetic, and imaging data to accurately forecast changes in cognitive functioning in PD patients. Methods: An artificial neural network was trained on baseline data to predict general cognitive status three years later. Model performance was evaluated using 5-fold stratified cross-validation. We investigated model interpretability using explainable artificial intelligence techniques, including Shapley Additive Explanations (SHAP) values, Group-Wise Feature Masking, and Brute-Force Combinatorial Masking, to identify the most influential predictors of cognitive decline. Results: The model achieved a recall of 0.91 for identifying patients who developed cognitive decline, with an overall classification accuracy of 0.79. All applied explainability techniques consistently highlighted baseline MoCA scores, memory performance, the motor examination score (MDS-UPDRS Part III), and anxiety as the most predictive features. Conclusions: From a clinical perspective, the findings can support the early detection of PD patients who are more prone to developing cognitive decline, thereby helping to prevent cognitive impairments by designing specific treatments. This can improve the quality of life for patients and caregivers, supporting patient autonomy.

## 1. Introduction

Parkinson’s disease (PD) is the second most common neurodegenerative disease [1], mainly characterized by the presence of motor symptoms such as bradykinesia, rigidity, resting tremor, and alterations of gait and posture [2]. Moreover, from the earliest stages of the disease, it is possible to detect the presence of non-motor symptoms, including sensory deficits, autonomic dysfunctions, sleep disturbances, behavioral and psychiatric disorders (such as depression, apathy, anxiety, and impulse control disorders), and cognitive impairments (e.g., [3,4]).

Focusing on cognitive impairments, the domains most affected are speed processing, attentive and executive functions, visuospatial abilities, memory, and learning (e.g., [5]). Moreover, impairments in the Theory of Mind and reward-based decision making may also occur [6,7,8]. Cognitive impairments can worsen as the disease progresses, being a risk factor for the development of dementia [9], even if the course of such impairments can be variable (some longitudinal studies reported that a portion of patients with mild cognitive impairments can remain stable over time and even present a reversal to normal cognition that, however, leads to a higher risk for developing future cognitive impairments; e.g., [10,11]).

It is well-established that the presence of cognitive impairments not only significantly affects the autonomy and quality of life of PD patients requiring a greater degree of clinical assistance but also increases the caregiver’s burden [12,13].

To date, multifactor possible pathophysiological causes have been hypothesized to be at the basis of cognitive impairments and dementia, such as the dopaminergic neuronal loss and the presence of Lewy bodies in cortical and limbic regions, the presence of tau and amyloid pathologies along with the presence of ε4 allele of the Apolipoprotein E (APOE), alterations in other neurotransmitter systems, early synaptic changes, inflammation, and mitochondrial dysfunction [10,14]. Moreover, gene mutations like a-synuclein (SNCA) or glucocerebrosidase (GBA) mutations are considered to be linked to a higher probability of developing a more rapid cognitive decline and an earlier onset of dementia in PD [10,15,16].

Concerning the variables linked to the presence of cognitive decline, the main possible risk factors that the literature highlights are usually the older age of the patient and of the PD onset, a longer duration of the disease, akinetic-rigid PD subtype and a higher severity of motor symptoms, the occurrence of hallucinations, reduced sense of smell, the presence of REM sleep behavior disorder, and the presence of depression (e.g., [16,17,18,19]). Moreover, the findings highlighted that baseline impairments in semantic fluency, visuospatial abilities, and memory-related functions may be linked to a higher risk of developing dementia compared to executive function deficits [16,20,21,22].

Given the heterogeneity of variables linked to cognitive decline, finding and confirming which risk factors are crucial in predicting the emergence of cognitive impairments (and the pre-dementia stages of cognitive impairment) in PD patients has become increasingly important in recent decades. This is pivotal for gaining an in-depth understanding of the underlying mechanisms of cognition in PD and for developing prevention strategies. However, the literature in this field has mostly focused on explanatory modeling rather than predictive modeling. While the former approach aims to explain relationships between variables by relying on interpretable statistical models, the latter leverages statistical models or data mining algorithms to foresee new or future observations. In other words, predictive modeling is oriented toward maximizing predictive accuracy.

Although some studies have adopted predictive approaches by developing machine learning models to forecast cognitive impairment in PD, several gaps remain in the literature. Most existing models have been trained on limited sample sizes and have primarily relied on clinical features and neuroimaging data [23]. To address this gap in the literature, the present study embraces a predictive approach with the aim of forecasting future changes in cognitive functioning among PD patients. A machine learning model, specifically an artificial neural network (ANN), was employed to accurately predict the 3-year follow-up cognitive state of individuals with PD (*N* = 618), based on a broad range of baseline variables (e.g., clinical, cognitive, genetic, and imaging features) identified according to previous literature and clinical availability of such measures. The choice to opt for ANNs was motivated by their ability to model complex, non-linear relationships and their widespread use in classification tasks. ANNs are particularly well-suited for handling large-scale data, and they also hold considerable potential for clinical applications, particularly for diagnostic purposes, making them a suitable choice for the present study [24,25]. Furthermore, given the clinical importance of understanding which variables drive model predictions, we implemented three complementary post hoc explainability techniques (i.e., SHAP analysis, Group-Wise Feature Masking, and Brute-Force Combinatorial Masking). These methods allowed us to assess the contribution of individual features and feature groups, helping to identify which baseline variables were most influential in predicting potential cognitive decline.

The contribution of this study to the literature is twofold. First, although ANNs hold promise for diagnostic applications, they have primarily been used to forecast the onset of PD, while the prediction of cognitive decline in PD has received comparatively little attention. Second, few existing studies have incorporated a broad spectrum of clinical data to forecast PD-related cognitive impairment and to identify the most relevant predictive features by leveraging explainable AI techniques. This study aims to address these gaps.

The paper is structured as follows. The next paragraphs summarize the relevant literature and outline the study’s objectives. Section 2 describes the methodology adopted, while Section 3 presents the findings. Section 4 discusses the results, and Section 5 provides the concluding remarks.

### 1.1. Machine Learning Techniques in the Context of Parkinson’s Disease

The adoption of machine learning algorithms in PD literature has notably increased in the last decade. Machine learning refers to a class of computational methods that enable systems to learn patterns from data and make predictions or decisions without being explicitly programmed. Machine learning has emerged as a promising tool in the clinical field for diagnostic purposes, as it can effectively handle multimodal data and large volumes of variables simultaneously [23]. In the PD literature, machine learning was mostly employed for diagnostic purposes based on different types of variables, such as motor, vocal, and imaging data (see [26] for a review). However, more recently, there has been a growth in the literature concerning the prediction of cognitive decline and dementia related to PD (see [23,27] for reviews). Likewise, the literature on the topic is also interested in identifying cognitive subtypes of PD patients by adopting clustering machine learning algorithms, which are aimed at identifying different subgroups within a population based on different configurations of cognitive variables (see [17] for a review).

Among the various machine learning methods, ANNs represent a powerful class of algorithms inspired by the structure and function of the human brain. ANNs form the foundation of deep learning, a subset of machine learning that involves multiple layers of interconnected processing units (neurons) capable of capturing complex hierarchical representations in large datasets. Despite their potential, the use of ANNs to forecast cognitive impairment in PD has received relatively little attention compared to their application in PD diagnosis. Indeed, to date, ANNs have been mainly employed for the prediction of PD (see [28] for a review). For instance, previous studies have used vocal data (e.g., [29]) or neuroimaging techniques (e.g., [30]) to forecast the evolution of PD. However, to date, only a few studies have implemented a predictive model for PD cognitive impairment based on ANNs [23]. For instance, Nguyen and colleagues [31] aimed to predict the scores on the Montreal Cognitive Assessment (MoCA)—which is one of the most used screening tools to investigate global cognitive functioning—of PD patients and healthy controls using magnetic resonance imaging (MRI) data as input variables. In the study, the authors developed a deep learning autoencoder model, namely a deep learning neural network model, and compared it with a logistic regression classifier, reporting a better performance of the autoencoder model. In a similar study [32], functional MRI (fMRI) data were used to distinguish between PD patients with normal cognition, mild cognitive impairment, and dementia. Choi and colleagues [33] developed instead a model based on FDG positron emission tomography (PET) data and trained on a sample of healthy individuals and patients with Alzheimer’s disease to also forecast the cognitive decline of PD patients reporting good performances. Finally, a study by Chung and colleagues [34] tested the performance of an ANN model in predicting PD-related cognitive impairment based on both blood-based biomarkers and clinical features. Overall, the literature still lacks an ANN-based model that integrates different data sources (e.g., clinical, cognitive, genetic, and imaging features) commonly used in clinical practice to predict cognitive decline in PD.

### 1.2. Aims

The present study aims to longitudinally predict the cognitive status of PD patients using a combination of cognitive and non-cognitive variables commonly employed in clinical practice, including cognitive and clinical assessments, DAT imaging data, and genetic data. To this end, we trained an ANN to forecast cognitive status at a 3-year follow-up starting from baseline data. Cognitive status was categorized as either “Cognitively Impaired” or “Cognitively Intact,” according to scores on the MoCA [35], a widely used screening tool for assessing global cognition in PD [36,37].

In addition, this study aims to identify the baseline variables that most strongly influence the model’s prediction of future cognitive change in PD. To ensure the robustness and consistency of the results, we implemented three complementary explainability techniques following ANN training and evaluation. These methods allowed us to estimate the relative importance of each input variable and to investigate whether the most influential predictors identified by the model align with findings previously reported in the clinical literature.

## 2. Materials and Methods

### 2.1. Sample

Data were retrieved from the CPP Integrated Parkinson’s Database. The CPP Integrated Parkinson’s Database initiative, funded by Parkinson’s UK and consortium membership organizations, was launched in October 2015 by the Critical Path Institute (CPath). Specifically, the Parkinson’s Progression Markers Initiative (PPMI) database was used (www.ppmi-info.org/data). Launched as an ongoing longitudinal, observational, multi-center study with open-source datasets, the PPMI began initial recruitment in 2010. It evaluates the progression of clinical and cognitive symptoms and the imaging, biological, and genetic data of PD patients. Data used in the preparation of this article was obtained on 21 January 2025, from the Parkinson’s Progression Markers Initiative (PPMI) database (https://www.ppmi-info.org/access-data-specimens/download-data), RRID: SCR_006431. For up-to-date information on the study, visit http://www.ppmi-info.org.

All participants obtained approval from an ethics committee before starting the study, written informed consent was collected from all participants, and the study procedures were conducted according to the principles of the Declaration of Helsinki. All the PD participants involved (recruited within 7 years of diagnosis as specified by the protocol) met the inclusion and exclusion criteria identified by the PPMI protocol. The study documentation is available online at https://www.ppmi-info.org/study-design/research-documents-and-sops. Additionally, we considered only PD patients who had a confirmed clinical diagnosis of PD with a 3-year follow-up assessment, as well as available MoCA data at follow-up.

After applying these inclusion criteria, a final sample of 618 PD patients was retained for analysis. Table 1 displays the baseline demographic and clinical characteristics of the study participants. The sample consisted predominantly of male patients (60.5%). The participants’ age at baseline ranged from 33 to 82 years (M = 61.84; SD = 9.65), with an average of 15.74 years of education (SD = 3.55). The mean MoCA score at baseline was 27, while the average score at follow-up was 26. The average MDS-UPDRS Part III score at screening was 20. Additionally, approximately 30% of the participants were under regular pharmacological treatment at the time of the baseline assessment. Additional information about the data used in the present study is available at https://www.ppmi-info.org/access-data-specimens/guidance-resources.

### 2.2. Measures

The MoCA [35], a widely used screening tool for assessing global cognitive state in PD [36], was used considering the total score from baseline to 3-year follow-up. Additionally, various clinical, cognitive, and imaging tools, together with genetic information, were considered.

The cognitive tests considered were the Benton Judgment of Line Orientation 15-item (split-half) version (JLO) for visuospatial function [38]; the Hopkins Verbal Learning Test—Revised (HVLT-R) for memory [39]; the semantic (animal) fluency test [40] and the Letter Number Sequencing (LNS) [41] for executive functions; and the Symbol-Digit Modalities Test (SDMT) for speed processing [42].

The neuropsychiatric and affective dimensions were assessed using the Geriatric Depression Scale (GDS-15) [43] for depressive symptoms and the State-Trait Anxiety Inventory (STAI) [44] for anxiety. Impulse control disorders were assessed using the short version of the Questionnaire for Impulsive-Compulsive Disorders in Parkinson’s Disease (QUIP) [45]. The items related to the Movement Disorder Society Unified Parkinson’s Disease Rating Scale (MDS-UPDRS) [46] Part I were also considered. The REM Sleep Behavior Disorder Screening Questionnaire [47] was used to assess the presence and clinical features of the REM sleep behavior disorder.

Other clinical data regarding disease severity were collected through the MDS-UPDRS Part II, assessing how the disease affects the patient’s daily activities; the MDS-UPDRS Part III, reporting the motor symptoms directly observed by the clinician during a neurological examination; and the Hoehn and Yahr (H&Y) scale, staging the global severity of the disease [48].

Dopamine transporter (DAT) imaging uptake parameters of bilateral caudate and putamen were also considered.

Genetic data such as APOE status and mutations such as LRRK2, GBA, VPS35, SNCA, PRKN, PARK7, and PINK1 were included.

Control variables were also considered, such as socio-demographics (i.e., gender, age, and education), participants’ medication status, and the presence of deep brain stimulation.

### 2.3. The Artificial Neural Network Model and Training

An ANN was trained to predict cognitive decline in PD patients using baseline clinical, neuropsychological, genetic, and imaging data. The input matrix consisted of standardized variables, while the output was a one-hot encoded binary label representing cognitive status (Cognitively Intact vs. Cognitively Impaired) at a 3-year follow-up. The target labels were derived from MoCA scores, binarized using a literature-based cut-off [49]: scores equal to or greater than 26 indicated preserved cognition, while scores below 26 indicated the possible presence of cognitive impairment.

In general, an ANN is a type of machine learning model inspired by the structure of the human brain. It is composed of layers of interconnected units (“neurons”) that transform input data through a series of mathematical operations. These layers enable the model to learn complex, nonlinear relationships between input variables and target outcomes [50].

During training, the ANN learns optimal weights, that is, the strengths of connections between neurons, by minimizing a loss function that quantifies prediction error. This is achieved through backpropagation, an algorithm that computes how much each weight contributes to the error and adjusts it accordingly. Specifically, backpropagation sends the error signal from the output layer backward through the network, layer by layer, allowing the optimizer to update each weight in the direction that reduces the overall error [51]. Through repeated iterations (epochs), the network refines these weights to improve its predictive accuracy. This process allows the ANN to progressively “learn” how to make better predictions from the input data.

To select the network architecture for this study, a grid search was performed across three hidden-layer depths (1, 2, or 3 layers) and three neuron counts per layer (7, 9, or 14). All models achieved comparable recall (≈0.90–0.94). However, the deeper or wider networks achieved their marginal recall gains by introducing more false positives. The single-hidden-layer network with 7 neurons matched the top recall while keeping errors in the Cognitively Intact class lowest, so we selected this parsimonious feed-forward design as the optimal balance of performance and interpretability. This choice could reduce the risk of overfitting and ensure clinically meaningful insights. In contrast, deeper networks, although powerful for very large datasets, can increase complexity and reduce interpretability, making them less suitable for smaller or moderately sized datasets and clinical applications [52].

**Figure 1:**
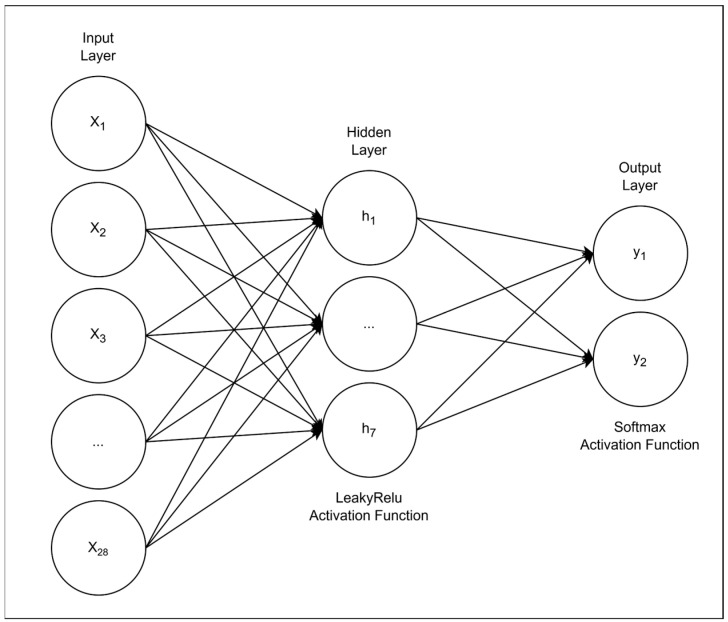
ANN architecture. The model receives 28 standardized baseline features as inputs, passes them through a single hidden layer of seven fully connected neurons with a leaky-ReLU activation, and outputs two soft-max units representing the probabilities of “Cognitively Impaired” and “Cognitively Intact,” respectively. Arrows denote dense, feed-forward connections.

Given the grid search results, the network architecture was implemented in Keras [53] and consisted of a single hidden layer with 7 units using the leaky ReLU activation function [54], and an output layer with two neurons using Softmax activation to produce a probability distribution over the two possible outcome classes [55]. Figure 1 provides a schematic overview of the implemented model architecture.

For binary classification purposes, the predicted probabilities were rounded using a threshold of 0.5, modally assigning patients to the Impaired class if the predicted probability for that class was equal to or greater than 0.5. Model training employed the Adam optimizer [56], a batch size of 8, and a maximum of 50 training epochs. Early stopping was applied based on validation loss to prevent overfitting [57].

Because fewer patients in the dataset developed cognitive impairment over the follow-up period, the classes were imbalanced. In such cases, traditional loss functions can bias the model toward the majority class. To address this, we used a focal cross-entropy loss [58], a loss function designed to give more importance to hard-to-classify cases, typically those in the minority class.

Focal loss reduces the contribution of easy examples and increases the weight of misclassified or difficult examples. It includes two key parameters:

• α (alpha): weights assigned to each class to balance their influence;
• γ (gamma): a focusing term that amplifies the penalty for misclassifications.

Focal loss is defined as

(1) $$FL\left(p_{t}\right)=-\alpha_{t}\left(1-p_{t}{)}^{\gamma }log(p_{t}\right)$$

where $p_{t}$ is the predicted probability for the true class, $\alpha_{t}$ is the class-specific weight, and γ is the focusing parameter. We used *α* = [0.5, 2.0] to upweight the Impaired class and *γ* = 1.5, prioritizing the model’s ability to learn from impaired cases.

Model evaluation was performed using 5-fold stratified cross-validation. The dataset was divided into five subsets (folds), ensuring that each fold maintained the original proportion of preserved and impaired cases. The model was trained on four folds and validated on the remaining one, repeating this process five times so that every participant appeared in a validation set exactly once [59].

From the five trained models, the one achieving the highest recall for the Impaired class was selected for the final evaluation. Recall (also referred to as sensitivity) is defined as the proportion of actual impaired cases that were correctly identified by the model:

(2) $$Recall=\left(True\;Positives\right)/\left(True\;Positives+False\;Negatives\right)$$

Recall is especially important in medical applications, where failing to identify at-risk individuals (false negatives) could have serious consequences [60]. By selecting the model with the highest recall for impaired patients, we prioritized clinical sensitivity, ensuring that patients likely to experience cognitive decline were correctly flagged, even at the cost of some false positives. The selected best-performing model was finally evaluated on the entire dataset to generate performance metrics.

To provide a broad view of model performance, we first report accuracy, defined as the proportion of correct predictions:

(3) $$Accuracy=\left(True\;Positives+True\;Negatives\right)/(True\;Positives+True\;Negatives+False\;Positives+True+False\;Negatives+False)$$

The model performance is also evaluated across all possible decision thresholds by constructing a ROC curve. For each threshold $\tau \;\in \;\left[0,1\right]$, we computed the true-positive rate (TPR) and false-positive rate (FPR) and plotted the TPR versus FPR.

The area under the ROC curve (AUC) was then calculated: AUC = 0.5 indicates chance-level discrimination, whereas AUC = 1.0 denotes perfect class separation.

Furthermore, precision and F1-score were computed to provide a more comprehensive assessment of predictive performance, particularly in the presence of class imbalance. Precision represents the proportion of predicted impaired cases that were actually impaired:

(4) $$Precision=\left(True\;Positives\right)/\left(True\;Positives+False\;Positives\right)$$

While recall emphasizes the identification of all true impaired cases, precision reflects the model’s ability to avoid false alarms. The *F*1-*score*, the harmonic mean of precision and recall, balances these two metrics into a single value:

(5) $$F1-score=2\times \left(Precision\times Recall\right)/\left(Precision+Recall\right)$$

### 2.4. Model Explainability

#### 2.4.1. SHAP Analysis

Shapley Additive Explanations (SHAP) is a post hoc interpretability method based on cooperative game theory, particularly the concept of Shapley values [61]. It quantifies the contribution of each feature to the model’s prediction by calculating how the predicted output changes when the feature is removed from (or added to) different subsets of input features. These marginal contributions are then averaged across all possible feature combinations, producing a SHAP value for each feature per prediction.

In this study, we applied SHAP to the trained ANN, approximating Shapley values through a combination of sampling and model evaluations. For interpretability, we extracted the SHAP values for the class of Cognitively Impaired and computed the mean absolute SHAP value for each feature across all patients.

This method allowed us to identify the most influential variables in the model’s decision making, providing a ranked list of features by their average importance. SHAP thus offered a fine-grained global view of how individual features influenced predictions.

#### 2.4.2. Group-Wise Feature Masking

While SHAP provides fine-grained insights at the level of individual variables, it does not directly assess the collective contribution of sets of related features, such as components derived from the same principal component analysis (PCA) grouping. To address this limitation, we implemented a group-wise masking procedure [62], in which features originating from the same PCA were treated as a single group.

Each group was iteratively masked by setting all its values to zero, effectively replacing the standardized features with their mean (due to prior standardization). This ensured architectural consistency, preserving input dimensionality and avoiding the need to retrain the network. After masking, we passed the modified dataset through the trained ANN and measured the change in recall (sensitivity) for the Impaired class. A larger drop in recall indicated that the masked group was more critical to the model’s ability to correctly identify at-risk individuals.

This technique offers an interpretable way to assess the importance of grouped features, particularly well-suited for evaluating composite constructs such as PCA-derived components. It complements the more granular SHAP analysis by highlighting the broader, domain-level influence of feature sets on model performance.

#### 2.4.3. Brute-Force Combinatorial Masking

To explore the interaction and cumulative effects of multiple variable groups, we extended the masking approach to a brute-force combinatorial framework. Specifically, we evaluated the impact of masking all possible combinations of up to 5 variable groups. For each combination, the selected groups were jointly set to zero, and the model’s recall on the Cognitively Impaired class was recalculated.

Formally, let $G=\left\{g_{1},g_{2},\ldots ,g_{20}\right\}$ be the set of variable groups (domain-specific components). For each subset S⊆G, where 1 ≤ ∣S∣ ≤ 5, all variables in groups S were set to zero, and the model’s predictions were re-evaluated. This procedure was repeated for all $\sum_{k=1}^{5}\frac{20}{k}=21700$ combinations.

The resulting recall scores were sorted to identify the combinations of groups whose absence most affected model performance.

This approach allowed us to identify not only which individual domains were influential, but also which combinations of domains were jointly critical for maintaining the model’s predictive performance following principles of subset-based explanation strategies [62]. For example, masking multiple mildly important groups together might reveal substantial performance degradation, highlighting synergistic effects not captured by SHAP or single-group masking.

**Figure 2:**
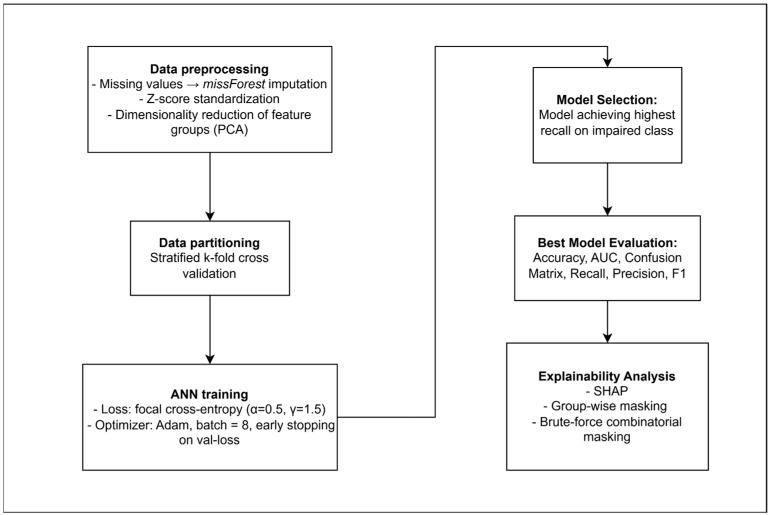
Flowchart summarizing all methodological steps.

A schematic overview of the full analysis workflow is provided in Figure 2.

## 3. Results

### 3.1. Descriptive Analysis and Data Preprocessing

The dataset included a wide set of clinical, cognitive, DAT imaging, and genetic features, each measured on different value scales. Each variable in the dataset had less than 20% missing values. To handle missing data, we applied the missForest algorithm in R [63], a nonparametric imputation method that uses random forests to iteratively predict and replace missing entries. This approach accommodates both continuous and categorical variables and does not rely on distributional assumptions.

To ensure that all variables contributed equally during model training, feature-wise standardization was applied using scikit-learn’s StandardScaler [64], which transforms each feature by subtracting its mean and dividing by its standard deviation. This step prevents features with larger numerical ranges from disproportionately influencing the training of the neural network.

As part of the preprocessing procedure, inter-feature correlations were examined, revealing several clusters of highly correlated variables, particularly within the neuropsychological and clinical assessment domains. High inter-feature correlation can introduce redundancy and reduce the effectiveness of model training by limiting the network’s ability to learn distinct patterns. To maintain clarity and conciseness in the manuscript, the full correlation matrix is provided as Supplementary Materials and is accessible via the Open Science Framework (OSF) (https://osf.io/nh72w/?view_only=478244609d1c47ada9c3b11646e66c44, accessed on 9 June 2025).

To reduce redundancy among correlated features, we performed principal component analysis (PCA) to feature groups with more than 5 variables, resulting in a smaller set of linearly uncorrelated components. For each feature group, we retained only the principal components with eigenvalues greater than 1.5, under the assumption that these components captured a meaningful proportion of variance while filtering out noise.

While the conventional Kaiser criterion suggests retaining components with eigenvalues greater than 1 [65], we adopted a more conservative threshold to ensure that only components accounting for a substantial amount of variance were retained. In particular, we adopted a cut-off of λ ≥ 1.5 because principal components with eigenvalues only slightly above 1 often capture marginal or noisy variance, especially in large, highly correlated clinical datasets. Restricting retention to components that clearly explain substantive variance helped reduce redundancy and lowered the dimensionality of the ANN input space, thereby improving the signal-to-noise ratio while maintaining predictive accuracy.

We emphasize that in this study, PCA was applied solely as a dimensionality-reduction step, without any interpretative purpose. The resulting principal components (PCs) are orthogonal, variance-capturing linear combinations of the original variables and should be regarded as technical constructs rather than theory-driven latent factors. The orthogonal principal components allow us to condense the highly correlated clinical and cognitive feature blocks into a smaller, noise-reduced set and to provide a more stable input space for the ANN.

**Figure 3:**
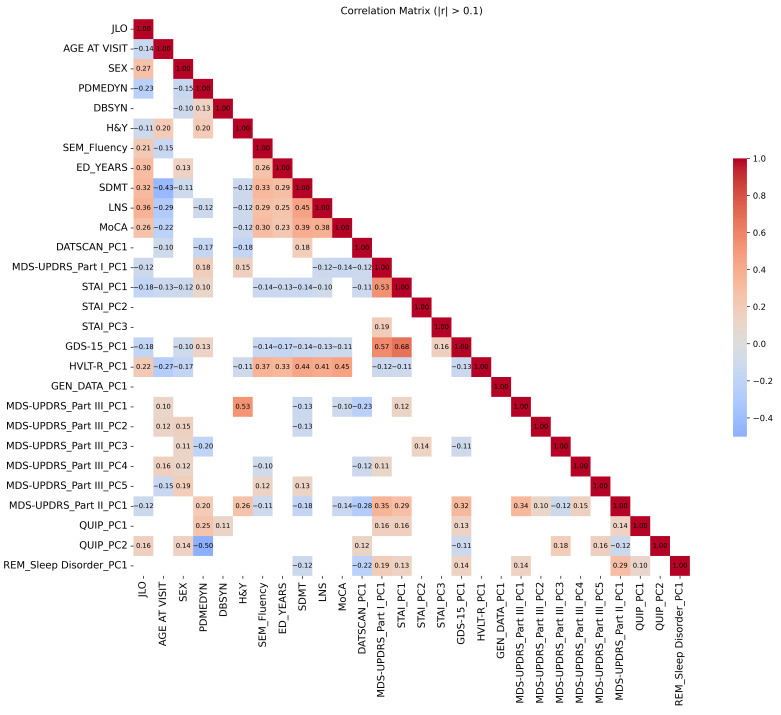
Correlation matrix for the final set of input variables used in the model training. In line with Cohen’s [66] effect size conventions, where r = 0.10 is considered a small effect, r = 0.30 a medium effect, and r = 0.50 a large effect, only correlations greater than r = 0.10 are shown.

Table 2 lists the original number of variables for each and the principal components retained based on the eigenvalue > 1.5 criterion. The initial set of 166 variables was reduced to 28 by applying PCA separately within the defined variable groups. Figure 3 shows the correlation matrix of the final variable set.

The resulting component scores replaced the original features for those groups in the final dataset used to train the ANN. This preprocessing step effectively preserved the most informative variance within each group while reducing the overall feature space, thereby mitigating overfitting risk and improving the model’s capacity to generalize.

As a preliminary step in modeling, we analyzed cognitive transitions to determine how many participants exhibited cognitive decline or remained stable over the follow-up period. Using the binary labels already defined based on the MoCA cut-off [49], we counted how many individuals shifted from the “Cognitively Intact” class to the “Cognitively Impaired” class, and vice versa, as well as those who remained in the same class.

Table 3 summarizes the transition distribution: 371 individuals remained stable and unimpaired (Cognitively Intact class), 92 were stable but impaired (Stable Impaired class), 100 converted from unimpaired to impaired (Conversion to Impaired class), and 55 reverted from impaired to unimpaired (Reversion to Cognitively Intact class). This distribution reveals a class imbalance, with Cognitively Impaired individuals at follow-up representing the minority class.

The four transition groups were not used for the network training but provide descriptive context to (i) illustrate the longitudinal heterogeneity of the cohort, (ii) highlight the resulting class imbalance, and (iii) allow post hoc inspection of how model errors distribute across clinically meaningful trajectories.

### 3.2. Model Performance

The ANN was trained for binary classification (Cognitively Intact vs. Cognitively Impaired) as reported in Section 2.3.

**Figure 4:**
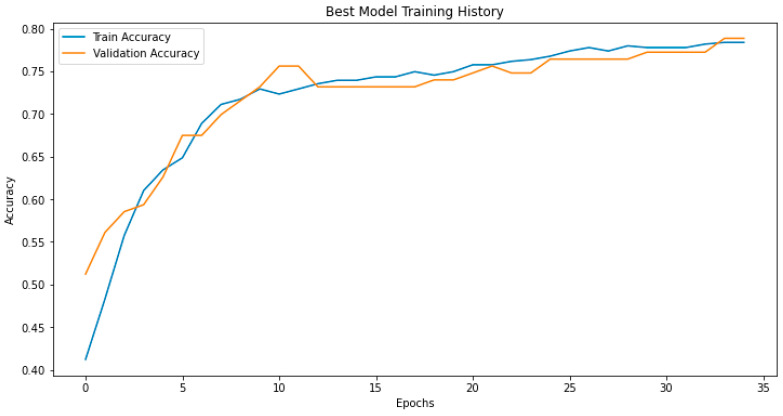
Training and validation accuracy across epochs for the best-performing model.

The best artificial neural network model across the five folds showed good performance in identifying patients who developed cognitive impairment. As illustrated in Figure 4, the model converged steadily during training, with minimal overfitting between the training and validation accuracy curves.

The network produced probabilistic outputs through a softmax activation function in the final layer, which assigns a probability to each class. For classification purposes, the predicted probability for the Impaired class was rounded using a threshold of 0.5 (i.e., patients with a predicted probability greater than or equal to 0.5 for impairment were classified as impaired).

**Figure 5:**
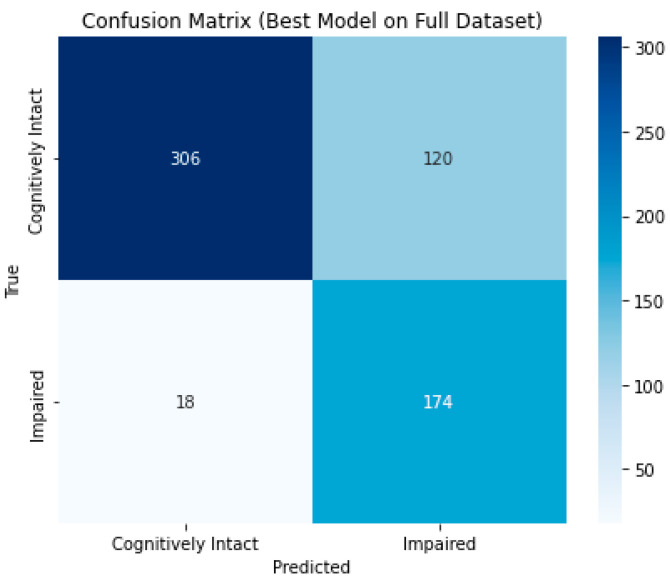
Confusion matrix for the best-performing model on the full dataset. The matrix displays the number of true and false predictions for each class.

According to the confusion matrix (Figure 5) and performance metrics (Table 4), the model correctly classified 174 out of 192 impaired patients, thus achieving a recall of 0.91 for the Cognitively Impaired class. This indicates high sensitivity in detecting individuals at risk of cognitive decline at follow-up.

**Figure 6:**
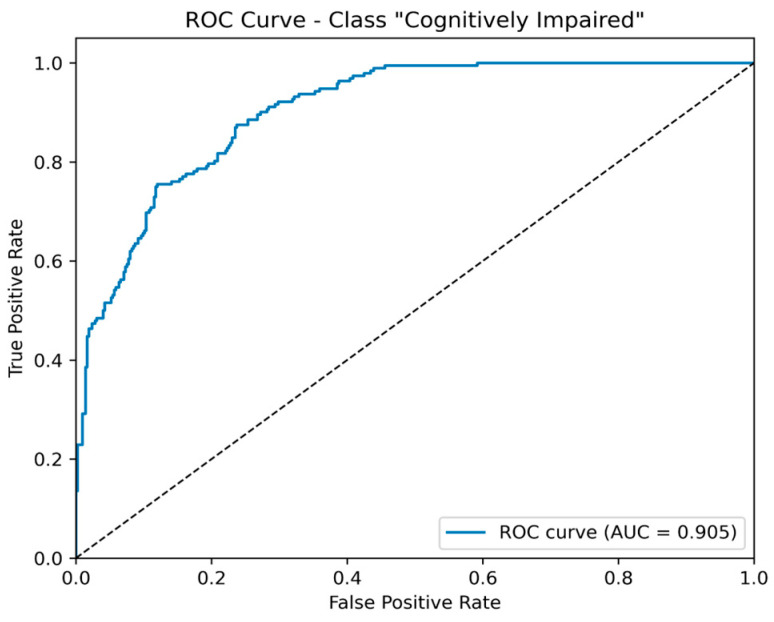
Receiver-operating characteristic (ROC) curve for the “Cognitively Impaired” class. The curve plots the true-positive rate against the false-positive rate across all probability thresholds; the area under the curve (AUC) is 0.905, indicating good discriminative capability.

The overall accuracy was 0.78, and the area under the ROC curve (AUC) reached 0.91, confirming good class separability. The corresponding ROC curve is displayed in Figure 6.

While precision for the Cognitively Impaired class was lower (0.59), this trade-off is consistent with the model’s prioritization of sensitivity over specificity, appropriate in clinical contexts, where failing to detect at-risk individuals is more detrimental than false positives. However, the F1-score for the Cognitively Impaired class was 0.72, reflecting a balanced compromise between recall and precision. For the Cognitively Intact class, the model achieved higher precision (0.94) and F1-score (0.82), though with a lower recall (0.72), suggesting that some individuals were misclassified as impaired, consistent with the results discussed before. This behavior reflects the model’s tendency to favor sensitivity, which aligns with the clinical objective of early identification of patients at risk for cognitive decline.

To gain a deeper understanding of model behavior, we examined prediction accuracy across the four cognitive classes: Cognitively Intact, Conversion to Impaired, Reversion to Cognitively Intact, and Stable Impaired (Table 5).

The majority of misclassifications occurred in the Cognitively Intact class, where 84 out of 371 individuals were incorrectly predicted as Impaired. Despite this, the model correctly identified 85% of patients who transitioned from Cognitively Intact to Impaired (Conversion to Impaired, n = 100), with only 15 misclassified, highlighting its ability to detect clinically meaningful deterioration.

The most challenging subgroup was the Reversion to Cognitively Intact class (n = 55), in which 36 individuals were incorrectly predicted, suggesting that fluctuating cognitive states may present ambiguous patterns for the model. Conversely, the model showed high specificity in the Cognitively Intact class, with only 3 out of 92 individuals misclassified, indicating reliable performance when cognitive impairment is persistent.

These findings reinforce the model’s clinical utility: its high sensitivity for conversion cases aligns with the goal of early identification of at-risk individuals, while most false positives occurred in cases with no decline, a preferable outcome when the cost of missing true converters is high.

### 3.3. Model Explainability

To gain insight into which features contributed most significantly to the model’s prediction of future cognitive impairment, we implemented three different explainability techniques: a SHAP analysis, a group-wise feature masking procedure based on recall change, and a brute-force group masking procedure.

#### 3.3.1. SHAP Analysis

**Figure 7:**
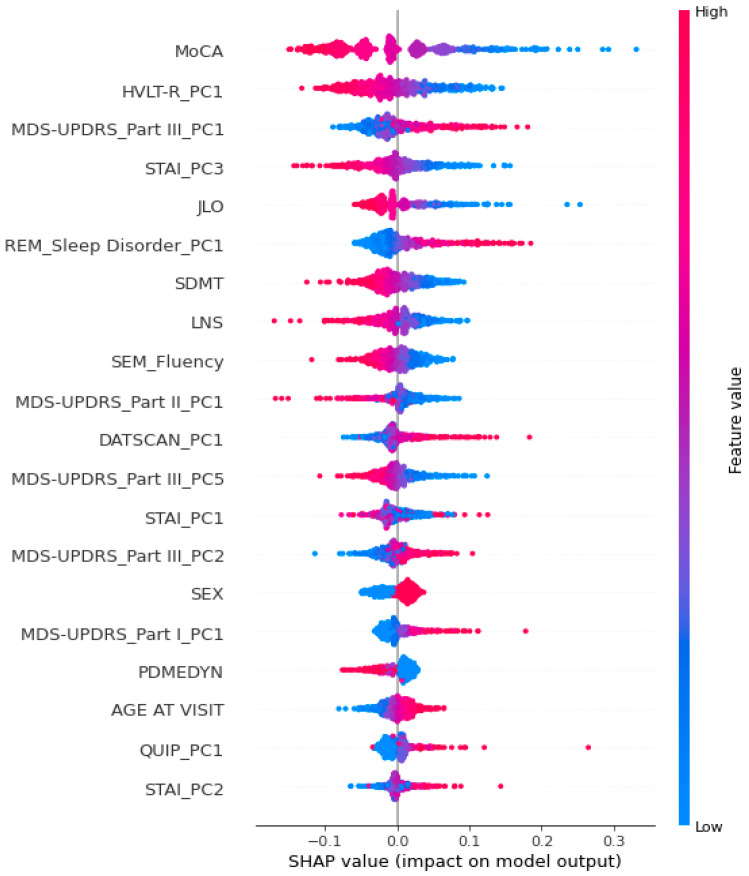
SHAP summary plot illustrating the contribution of each feature to the model’s prediction of cognitive impairment. Each point represents a SHAP value for a patient; colors indicate the original feature value (red = high, blue = low). Features are ranked by their mean absolute SHAP value, with top-ranked variables contributing the most to the model’s decisions. Labels ending in “_PCn” refer to the n-th principal component extracted from the corresponding neuropsychological or clinical assessment using PCA.

The SHAP summary plot (Figure 7) displays the global feature importance based on the average magnitude of each variable’s contribution to the model’s output. Features are ranked by their mean absolute SHAP value, and the color gradient indicates the original feature value (from low to high) for each data point.

As shown in the SHAP summary plot (Figure 7), among all predictors, baseline global cognitive performance measured by the MoCA emerged as the most impactful variable, with lower scores consistently pushing the prediction toward the Impaired class. Additionally, several principal components derived from the cognitive, motor evaluation, and affective domains ranked among the most influential predictors. Notably, components such as HVLT-R_PC1 (verbal memory), MDS-UPDRS_Part III_PC1 (severity of motor symptoms), and STAI_PC3 (anxiety) were among the highest contributors to the model’s predictions. Although PCA components are derived through data-driven variance maximization rather than theory-driven constructs, their importance in the model highlights the clinical relevance of the domains they represent. However, given the PCA data-driven approach, we must prevent an explicit theoretical interpretation of the meaning underlying the individual components.

It is important to note that the mean absolute SHAP over all observations is an intuitive measure of average prediction shift, but this quantity is not directly comparable to classical statistical effect sizes (e.g., Cohen’s d or η^2^), which are grounded in variance explained in the observed target. To obtain a performance-oriented analogue, we additionally report Δ-recall produced by group-wise and combinatorial masking. These recall drops indicate how strongly overall model sensitivity depends on each domain (or combination of domains) and thus serve as practical “effect-size-like” metrics for our predictive task.

#### 3.3.2. Group-Wise Feature Masking

To further evaluate the impact of specific variable groups on the model’s predictive ability, we implemented a Group-Wise Feature Masking procedure based on recall. Since individual PCA components are not directly interpretable from a theoretical standpoint, we grouped related components to enable a more domain-specific interpretation of their collective influence on model performance.

**Figure 8:**
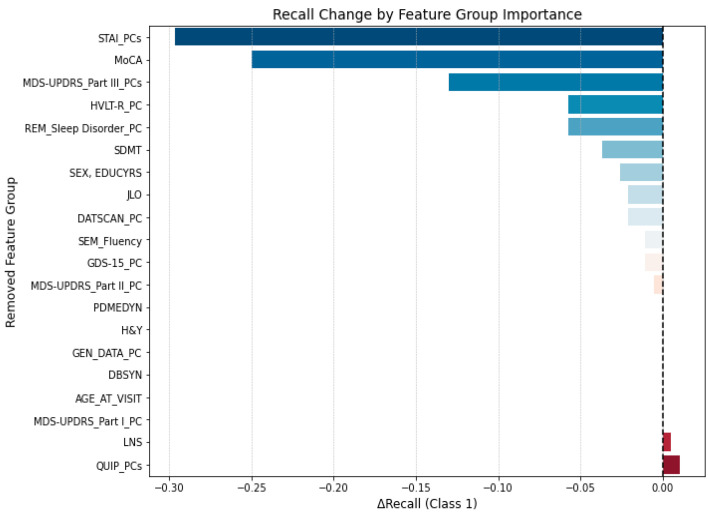
Impact of group-wise feature masking on model recall for the impaired group. Each bar represents the change in recall (∆Recall) observed when the corresponding variable group was set to zero (i.e., masked). Larger negative values indicate greater importance of the variable group for model sensitivity. Shades of blue indicate a drop in recall when the variable group is removed (darker blue = larger decrease), while shades of red indicate an increase (darker red = larger gain). The dashed vertical line denotes ∆Recall = 0.

In this approach, we iteratively set to zero predefined groups of related features, either clinical domains or PCA-derived components, and we measured the resulting change in recall for the Impaired class. Figure 8 shows the recall drop for each variable group, sorted by impact. Larger negative values indicate a greater drop in recall and, therefore, a stronger contribution of that feature group to the model’s sensitivity.

The most substantial decrease in recall occurred when removing the anxiety-related features (STAI_PCs), resulting in a ∆Recall of −0.30. This was followed by the MoCA score (−0.25), motor score (MDS-UPDRS_Part III components; −0.13), and verbal memory principal component (HVLT-R_PC, −0.06). These findings support the results of the SHAP analysis, reinforcing the importance of cognition, affective symptoms, and motor symptomatology in predicting future cognitive impairment.

#### 3.3.3. Brute-Force Feature Elimination Procedure

To examine the collective influence of multiple variable sets, we conducted an exhaustive brute-force analysis in which all possible combinations of up to five feature groups were masked, and their effect on recall was evaluated. The brute-force combinatorial masking analysis further highlighted key feature groups contributing to model performance by evaluating the impact of simultaneously masking up to five variable sets. Among the 10 lowest-performing combinations, recall values dropped as low as 0.547, indicating a substantial loss of predictive sensitivity when specific feature sets were set to zero. Most notably, combinations, including the MoCA score, the HVLT-R, the MDS-UPDRS Part III, and the STAI scale, were consistently associated with the largest reductions in recall. This aligns with the results from both SHAP and group-wise masking, reinforcing the importance of these domains in identifying patients at risk for cognitive decline. These findings suggest that the model’s predictive performance depends not only on individual variables but also on the combined presence of interrelated cognitive, motor, and affective features, highlighting the benefit of multimodal input in clinical risk modeling.

## 4. Discussion

The present study showed a novel approach to investigate and identify pivotal variables that can longitudinally predict general cognitive decline in PD patients in a 3-year follow-up. To do so, we considered variables inferred from tools commonly used in clinical practice, such as cognitive tests assessing the main cognitive domains (i.e., visuospatial functions, memory, executive functions, and speed processing), self-report questionnaires for deepening the neuropsychiatric and affective dimensions, neurological scales widely used to evaluate the severity of the disease, DAT imaging data, and genetic data.

ANN models, like the one implemented in this study, are particularly well suited for modeling complex, nonlinear relationships across multiple data types, making them ideal for clinical scenarios where cognitive decline emerges from the interplay of manifold and different factors. Their flexibility allows the integration of heterogeneous data into a unified predictive model.

Furthermore, unlike traditional classification approaches that operate cross-sectionally, our work focuses on longitudinal prediction. Indeed, the ANN was trained to identify patients who would develop cognitive decline over a 3-year follow-up period, using only baseline data. This predictive framing represents a substantial shift in both clinical utility and methodological design, as it aims to identify patients at higher risk, supporting the clinician in early detection before the onset of measurable impairment.

Importantly, the model was trained to output class probabilities rather than hard class labels. This probabilistic output provides clinicians with a confidence estimate for each patient, allowing nuanced interpretation beyond binary classification. For instance, a moderate predicted probability of possible impairment may prompt early strategic clinical decisions, even if it falls below a decision threshold.

Findings revealed that the model achieved a high sensitivity (0.91) in detecting patients at risk of presenting cognitive decline at a 3-year follow-up and a lower but acceptable sensitivity (0.72) in identifying patients with a cognitively intact profile at follow-up. The model’s performance, particularly its high sensitivity for the Impaired class (0.91), is consistent with or exceeds the sensitivity ranges reported in recent reviews of prognostic models for cognitive decline in PD [23].

Although the model’s precision for the Impaired class was 0.59, resulting in some cognitively intact individuals being misclassified as impaired, this outcome reflects an intentional trade-off that prioritizes early detection by maximizing sensitivity. This strategy is based on the idea that in clinical contexts, the ability to identify individuals at risk of cognitive decline is often considered more important than minimizing false positives, as early intervention opportunities may otherwise be missed. For instance, patients may lose opportunities to engage in rehabilitation or treatment programs, to receive targeted preventive recommendations and guidelines to follow in their everyday lives or to undergo further examinations or specific monitoring (e.g., [36,67]). It can lead to significant consequences not only for the patients but also for their families and society as a whole. Accordingly, the model’s performance supports its potential utility in aiding the early identification of patients at risk for cognitive deterioration.

The results achieved by the ANN model are consistent with those of other studies aimed at forecasting cognitive impairment related to Parkinson’s disease [23,27]. Notably, the most relevant predictive variables align with those identified by Almgren and colleagues [68], which used the same dataset from the PPMI, although with a smaller sample size, different input variables, and a different machine learning algorithm (i.e., Support Vector Regression). In that study, the most significant features were baseline MoCA scores and psychological scales (i.e., STAI and GDS). However, it is important to note that the findings are not fully comparable, as the present study included a different set of input variables compared to Almgren and colleagues’ one [68].

### 4.1. A Focus on the Crucial Variables Identified by the Model

It is interesting to observe that the variables that emerged as most significant to the model’s prediction are consistent among the SHAP analysis, the Group-Wise Feature Masking procedure, and the brute-force analysis. These variables include the baseline MoCA scores, memory performance, MDS-UPDRS Part III scores, and anxiety levels. Regarding the importance of the baseline MoCA score for the model, this may seem evident, as it is also the tool selected as the outcome measure at follow-up. Nonetheless, this result supports existing evidence indicating that the MoCA score has a predictive value for progressive cognitive decline and is appropriate for assessing cognitive functioning in PD (e.g., [36,37]).

Moreover, concerning cognitive variables, it is interesting that memory plays a crucial role in predicting cognitive decline in PD, confirming previous studies that investigated such a cognitive variable [19,69] through the HVLT-R as well. From a recent review analyzing cognitive tests useful to detect impairments in PD, HVLT-R is highlighted as a reliable measure and a sensitive tool for detecting the progression to cognitive deterioration [70], and our findings can support such evidence. Moreover, the crucial role of memory performance in predicting cognitive decline in PD patients can find possible explanations in the “dual syndrome hypothesis” [22]. According to it, patients who mainly present executive dysfunctions—that are hypothesized to be primarily associated with changes in dopaminergic pathways—are assumed to be less likely to progress to PD dementia (consistent with our results, in which executive function tests showed a lower contribution to the model prediction compared to memory performance). In contrast, those showing memory-related and visuospatial impairments—probably mainly resulting from cholinergic deficits involving frontal and temporal regions—are at greater risk for rapid cognitive decline and progression to dementia [16,20,22].

This hypothesis is widely considered, even if further studies are needed to better understand and account for (i) the greater complexity of the disease progression, involving both distinct and interacting impairments of the catecholaminergic and cholinergic systems (e.g., [20]); (ii) the cognitive decline in PD, which is the result of a complex process (e.g., [10]), the possible presence of coexisting pathologies, such as concomitant Alzheimer’s disease-like pathology, which may contribute to a more rapid cognitive decline in memory and visuospatial domains; and (iii) the possible age differences between these two cognitive profiles, as in the literature there is evidence that a prominent memory-related impairment profile can be characterized by a higher mean age of the patients (and also higher mean age at PD onset) compared to the predominant executive dysfunction profile [17]. However, recent research, considering PD patients characterized by a heterogeneous disease duration varying from de novo to more than 20 years, better highlights the involvement of different neural substrates underlying these two possible distinct cognitive patterns, supporting our results. Specifically, it confirmed that performance related to attention and executive function domains ( which the authors assessed through the SDMT and Trail Making Test) was related to the caudate uptake data obtained from DAT imaging (confirming the crucial role of dopamine and the frontostriatal structures in such abilities), while performance related to memory and visuospatial domain (assessed through the HVLT-R and the JLO test, respectively) was related to regional cortical thickness in the left frontotemporal and right frontal lobes acquired through MRI data [21], highlighting the role of cortical disturbances in developing difficulties in such abilities. Moreover, a comparison between cognitively intact PD patients and patients with mild cognitive impairment showed a significant cortical degeneration in the second group, predominantly affecting the left-dominant bilateral frontal and anterior temporal regions [21]. Such results can support the link between a suboptimal performance in memory (and visuospatial domain, which also emerged from SHAP analysis in our model) and a higher risk of developing cognitive decline, highlighting the importance of including these abilities in cognitive assessments and considering with special attention patients who show related difficulties.

In line with the previous literature, the MDS-UPDRS Part III should also be considered, as it emerged as another crucial variable in the prediction of our model, according to previous evidence (e.g., [19,71,72,73]). Thus, such a result can support the assumption that in PD, motor impairment appears to be closely linked to cognitive dysfunctions [74].

The same goes for anxiety; although some studies have reported significant associations between anxiety scores and either performance in specific cognitive domains [75] or MoCA scores [72,73], the number of studies specifically addressing these relationships in PD patients remains limited. Given that anxiety and depression frequently co-occur in PD, at rates higher than those observed in the general population [76], and previous studies found a link between depressive symptomatology and cognitive impairments (e.g., [18,72,73]), it was a surprising finding in anxiety, but not depression, a strong predictor in our model. The GDS-15, which is widely used to assess the presence of depressive symptoms, was used. In this way, highlighting the crucial role of anxiety in our model and the fact that so far, only a few studies have considered it when investigating cognition, further studies are needed to better understand the underlying mechanisms.

The severity of motor symptoms and anxiety levels, together with memory-related impairments, can suggest and support assumptions that cognitive decline in PD may reflect the involvement not only of the dopaminergic pathways but also of the non-dopaminergic systems, where a depletion of noradrenergic input to the cortex and cholinergic impairments may interact (e.g., [20,72,77]).

### 4.2. Further Lines of Research to Deepen Cognitive Functioning in Parkinson’s Disease

An interesting point that goes beyond the aims of the present study but that deserves further research is represented by the presence of the Reversion to Cognitively Intact class, which was composed of a limited number of patients and was the most challenging to predict for our model. So far, a limited number of studies have deepened that specific class, but the literature that explored such an issue highlighted an increased risk for further developing cognitive impairment or dementia in such patients [10,11,78]—with a similar trend also in populations without a diagnosis of PD [79,80]—compared to those who remain with a stable cognitive integrity. This may be a possible explanation for the higher rate of misclassifications obtained for this group by the model. In PD, some hypotheses have been made concerning this fluctuating cognitive state, such as that these patients initially benefited from possible interventions. For instance, effective and tailored cognitive training can be beneficial for specific cognitive functions [11]. Moreover, the initiation (or subsequent adjustments) of dopaminergic therapy can lead to cognitive changes, where executive functions mainly relying on the dorsolateral circuit typically benefit from it (e.g., [81]), possibly leading to an increase in the scores of tests assessing global cognitive functioning. Furthermore, such a reversion can be considered as belonging to non-motor fluctuations (which are common in PD patients [82]), or it may even mirror a measurement error (including a possible learning effect) rather than a real cognitive improvement, especially in patients whose MoCA scores are near the cut-off level [11,78]. In this way, further studies that consider larger samples of “reverters” and that monitor them over longer periods and in a more in-depth manner would be useful to better understand the phenomenon.

Another direction for future work involves building new predictive models based on the most relevant domains identified through explainability analyses. These further models would include all original variables from each domain (e.g., individual questionnaire items), rather than using aggregated or dimensionally reduced representations. Since these domains have already been refined through prior analysis, this approach would allow for deeper granularity in interpretation, making it possible to identify which specific part of each assessment contributes most to the prediction of future cognitive decline.

### 4.3. Limitations

The study has some limitations. First of all, to assess the cognitive status and accordingly classify patients, Level I criteria were used, where possible impairment is generally found based on a tool assessing global cognitive abilities and validated for being administered in PD patients, such as the MoCA [37]. In this way, Level I criteria provide less diagnostic certainty than Level II criteria, which recommend a formal, comprehensive neuropsychological assessment encompassing a minimum of two tests for each of the five main cognitive domains (e.g., attention and working memory, executive functions, language, memory, and visuospatial abilities). Evidence of impairment should be observed on at least two tests, either within the same domain or across different domains [37]. Unfortunately, comprehensive assessment was not available for all patients, as it can sometimes occur in daily clinical visits, and further studies can deepen and confirm our results by categorizing patients based on a more extensive cognitive evaluation. Accordingly, we were prevented from making comparisons between possible different impairment profiles (e.g., amnestic or non-amnestic ones). Future studies should consider distinct cognitive profiles for impaired groups to better understand possible differences in the follow-up outcomes.

Moreover, as in other studies in the literature investigating cognitive functioning, we were not able to classify more in-depth patients belonging to the Cognitively Impaired class—for instance, dividing those who scored 25–21 from those who scored below 21 MoCA points [49]—which would have resulted in having two groups with a strongly imbalanced number of cases. However, in line with our aims, we considered the MoCA score as a binary variable adopting a cut-off of 26 for cognitively intact status, as scores below 26 have been highlighted as highly predictive of progressive cognitive decline [36].

Furthermore, to align with our aims of employing clinically feasible and easily implementable data for monitoring patients with PD in clinical settings, we restricted our analysis to basic clinical, cognitive, main genetic data and mutations and DAT imaging data.

We excluded more invasive or less routinely collected measures, such as cerebrospinal fluid biomarkers and advanced neuroimaging data (e.g., resting-state fMRI), mainly due to both their limited availability in clinical settings and the significant proportion of missing values in the available data. Disease duration was also not included in the present analyses or in other sections of the paper (e.g., the descriptive table) because this information was unavailable for a large number of participants. However, according to the study protocols, the patients were enrolled within seven years of diagnosis.

## 5. Conclusions

The present findings are in line with previous studies that have separately analyzed the examined variables and/or used different analytical methodologies, (i) highlighting the complexity of the mechanisms underlying cognitive decline in PD and (ii) confirming the importance of considering diverse types of variables (including cognitive and non-cognitive data) in both clinical and experimental settings to better identify patients who may be more prone to developing cognitive decline.

Our methodological innovation—which applies rigorous explainability analyses to a longitudinal ANN trained on real-world, clinically relevant data—represents a meaningful advancement over prior research. Our findings not only confirm the importance of well-established predictors such as the baseline MoCA scores but also highlight the importance of combining data from cognitive tests with motor examination information and anxiety levels, as critical contributors to predicting cognitive decline in PD. The fact that these variables emerged as key features across all interpretability methods suggests that their relevance is not an artifact of a single analytical approach, but a robust signal captured by the model through different perspectives. This convergence strengthens the case for their inclusion in early cognitive risk assessments and demonstrates the added value of multimodal, explainable deep learning approaches in clinical neuroscience. In this way, the model functions as a decision-support tool by flagging patients who may otherwise be overlooked in the early stages of disease progression.

## Figures and Tables

**Table 1:** Baseline socio-demographic and clinical characteristics of the sample.

Variable	
Sex:	
Men	374 (60.5%)
Women	244 (39.5%)
Age at visit	M = 61.84, SD = 9.65
Years of education	M = 15.74, SD = 3.55
Pharmacological treatment:	
On regular treatment	193 (31.2%)
Without treatment	425 (68.8%)
MoCA score (baseline)	M = 26.92, SD = 2.51
MoCA score (3-year follow-up)	M = 26.41, SD = 3.29
MDS-UPDRS Part III	M = 20.35, SD = 9.23
Hoehn & Yahr staging:	
Stage 1	232 (37.5%)
Stage 2	377 (61%)
Stage 3	9 (1.5%)

Note. M = mean; SD = standard deviation.

**Table 2:** Summary of variable groups included in our model, including variables reduced via PCA.

Variable Groups	Original Number of Variables	Number of Components
Socio-demographic data:		
SEX	1	-
ED_YEARS	1	-
AGE AT VISIT	1	-
Clinical data:		
PDMEDYN	1	-
DBSYN	1	-
MDS-UPDRS_Part I	6	1
MDS-UPDRS_Part II	13	1
MDS-UPDRS_Part III	33	5
H&Y	1	-
Cognitive data:		
MoCA	1	-
JLO	1	-
HVLT-R	7	1
SDMT	1	-
LNS	1	-
SEM_Fluency	1	-
Neuropsychiatric and affective data:		
GDS-15	15	1
STAI	40	3
QUIP	13	2
REM_Sleep Disorder	12	1
Neuroimaging data:		
DATSCAN	6	1
Genetic data:		
GEN_DATA	10	1

Note. SEX—sex; ED_YEARS—years of education; AGE AT VISIT—age at visit; PDMEDYN—PD medication use (Y/N); DBSYN—deep brain stimulation (Y/N); MDS-UPDRS I–III—Movement Disorder Society-Unified Parkinson’s Disease Rating Scale Parts I–III; H&Y—Hoehn & Yahr stage; MoCA—Montreal Cognitive Assessment; JLO—Benton Judgment of Line Orientation; HVLT-R—Hopkins Verbal Learning Test—Revised; SDMT—Symbol Digit Modalities Test; LNS—Letter-Number Sequencing; SEM_Fluency—semantic fluency; GDS-15—Geriatric Depression Scale; STAI—State-Trait Anxiety Inventory; QUIP—Questionnaire for Impulsive-Compulsive Disorders in PD; REM_Sleep Disorder—REM sleep behavior disorder; DATSCAN—dopamine transporter imaging; GEN_DATA—genetic data (APOE status and LRRK2, GBA, VPS35, SNCA, PRKN, PARK7, and PINK1 mutations).

**Table 3:** Number of patients in each cognitive category, based on changes in cognitive status between baseline and follow-up.

Class	Count
Cognitively Intact	371
Stable Impaired	92
Conversion to Impaired	100
Reversion to Cognitively Intact	55

**Table 4:** Model classification performance.

Group	Count	Precision	Recall	F1	Accuracy	AUC
Cognitively Intact	426	0.94	0.72	0.82	-	-
Cognitively Impaired	192	0.59	0.91	0.72	-	-
Overall	618	0.78	0.81	0.79	0.78	0.91

**Table 5:** Misclassified analysis.

Class	Count	Misclassified
Cognitively Intact	371	84
Stable Impaired	92	3
Conversion to Impaired	100	15
Reversion to Cognitively Intact	55	36

## Data Availability

Data used in the preparation of this article were obtained from the Parkinson’s Progression Markers Initiative (PPMI) database (www.ppmi-info.org/data). Additional materials presented in this study are included in the article/Supplementary Materials. Further inquiries can be directed to the corresponding author.

## Supplementary Materials

Additional materials, including the full correlation matrix and Python scripts for model training and explainability analysis, are available from the OSF repository at https://osf.io/nh72w/?view_only=478244609d1c47ada9c3b11646e66c44.

## Abbreviations

The following abbreviations are used in this manuscript:

ANN	Artificial neural network
APOE	Apolipoprotein E
DAT	Dopamine transporter
fMRI	Functional magnetic resonance imaging
GBA	Glucocerebrosidase
GDS-15	Geriatric Depression Scale
H&Y	Hoehn and Yahr
HVLT-R	Hopkins Verbal Learning Test—Revised
JLO	Benton Judgment of Line Orientation
LNS	Letter Number Sequencing
MDS-UPDRS	Movement Disorder Society Unified Parkinson’s Disease Rating Scale
MoCA	Montreal Cognitive Assessment
MRI	Magnetic resonance imaging
PC	Principal component
PCA	Principal component analysis
PD	Parkinson’s disease
PET	Positron emission tomography
SDMT	Symbol-Digit Modalities Test
SHAP	Shapley Additive Explanations
SNCA	a-synuclein
STAI	State-Trait Anxiety Inventory
QUIP	Questionnaire for Impulsive-Compulsive Disorders in Parkinson’s Disease
